# Association of Sex Hormones and Sex Hormone-Binding Globulin Levels With Bone Mineral Density in Adolescents Aged 12–19 Years

**DOI:** 10.3389/fendo.2022.891217

**Published:** 2022-05-20

**Authors:** Ke Xu, Yicheng Fu, Buzi Cao, Mingyi Zhao

**Affiliations:** ^1^ Department of Pediatrics, The Third Xiangya Hospital, Central South University, Changsha, China; ^2^ Xiangya School of Medicine, Central South University, Changsha, China; ^3^ Department of Pediatrics, Wuhan University Renmin Hospital, Wuhan University, Wuhan, China; ^4^ Medical School, Hunan Normal University, Changsha, China

**Keywords:** sex hormones, testosterone, estradiol, sex hormone-binding globulin, adolescents, bone mineral density

## Abstract

**Background:**

Sex hormones are recognized to play a significant role in increasing bone mineral density (BMD) and promoting bone maturation during adolescence. The purpose of our study was to use a database with large population data to evaluate the association of BMD with sex hormones (including testosterone and estradiol) and sex hormone-binding globulin (SHBG) in adolescent boys and girls aged 12–19 years.

**Methods:**

The data for our study were taken from the National Health and Nutrition Examination Survey 2013-2016, and we used weighted multiple linear regression models to assess the relationship between testosterone, estradiol, and SHBG and total BMD. We use weighted generalized additive models and smooth curve fitting to discover underlying nonlinear relationships.

**Results:**

A total of 1648 teenagers (853 boys, 795 girls) were selected for the final analysis. In boys, testosterone and estradiol levels were positively associated with total BMD, whereas SHBG levels were negatively associated with total BMD after adjusting for covariates [P < 0.05; 95% confidence interval (CI)]. In addition, there was a point between estradiol and total BMD, after which the positive correlation between estradiol and total BMD was relatively insignificant in boys. In girls, there was a positive association between estradiol and total BMD (P < 0.05; 95% CI), but there was no significant association between the testosterone (β 0.0004; 95% CI -0.0001 to 0.0008) or SHBG (β -0.0001; 95% CI -0.0002 to 0.0001) levels and total BMD. We also found an inverted U-shaped association between testosterone and total BMD with the inflection point at 25.4 ng/dL of testosterone.

**Conclusions:**

We found differences in the association of sex hormones with total BMD in boys and girls. Based on our findings, an appropriate increase in serum testosterone levels may be beneficial for skeletal development in girls because of the inverted U-shaped relationship (with the inflection point at 25.4 ng/dL of testosterone), and a high testosterone level might be detrimental to BMD. Furthermore, keeping estradiol levels below a certain level in boys (24.3 pg/mL) may be considered.

## Introduction

Osteoporosis is a major public health issue that threatens the health of millions of people globally (1). It is characterized by very low bone mass and destruction of the microstructure of the bone, which greatly increases the risk of fracture. It is well known that adolescence is an important period of bone growth, development, and maturation. During adolescence, bone builds up and grows rapidly. By the end of puberty, the bone mass approaches approximately 90% of the adult peak bone mass (2). Obtaining a higher bone mineral density (BMD) during adolescence is essential for bone mass gain and bone maturation to achieve a higher peak bone mass for prevention of osteoporosis in old age (3).

Sex hormones and growth hormone (GH) are the main determinants of BMD, and sex hormones are recognized to play a significant role in increasing BMD and promoting bone maturation during adolescence. Estrogens modulate changes in bone geometry during puberty and stimulate periosteal bone deposition while inhibiting cortical bone resorption (4). Testosterones increase bone diameter by increasing periosteal apposition (5). A marked increase in sex hormone levels occurs during puberty, as boys and girls develop differences in their skeletal development owing to the differences in sex hormone levels in their bodies.

Sex hormone-binding globulin (SHBG) is synthesized by the liver and released into the bloodstream, where it regulates the bioavailability of sex hormones by binding to them (6). Studies have thus far evaluated the association of SHBG with BMD in adults, postmenopausal women, and older adults (7–9). However, the association in adolescent boys and girls remains inconclusive. The purpose of our study was to use a database with large population data to evaluate the association of BMD with sex hormones (including testosterone and estradiol) and SHBG in adolescent boys and girls aged 12–19 years.

## Materials and Methods

### Data Source

The National Health and Nutrition Examination Survey (NHANES) is a large, population-based cross-sectional survey aimed at gathering information on the health and nutrition of the general American population. The National Center for Health Statistics (NCHS) authorized and conducted these surveys.

**Figure 1:**
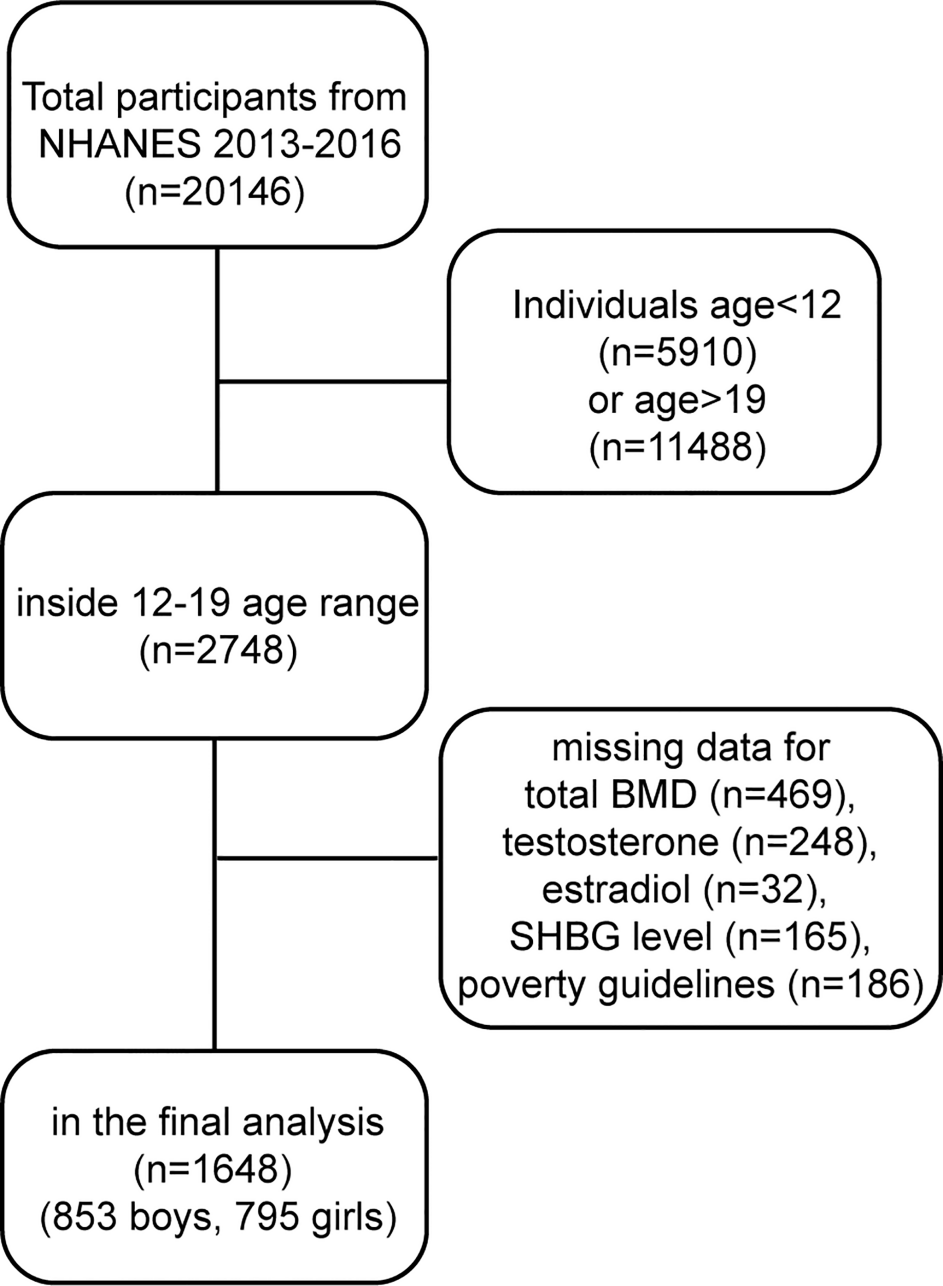
Research flow chart.

The data for our study were gathered from the NHANES 2013-2016. The population of this study was restricted to adolescents aged 12–19 years. After filtering according to the flow chart (**Figure 1**), 1648 participants (853 boys, 795 girls) aged 12–19 years were selected for the final analysis.

The NHANES was approved by the NCHS Ethical Review Board, and informed consent was obtained from all participants. For those under the age of 18, informed consent was provided by their parents/guardians and those aged 18 and over provided their own consent.

### Study Variables

The exposure variables of this study were testosterone, estradiol and SHBG levels. Testosterone and estradiol were measured by isotope dilution liquid chromatography tandem mass spectrometry (ID-LC–MS/MS). SHBG was measured based on its reaction with immuno-antibodies and chemo-luminescence. The outcome variable was total BMD, which was measured using dual-energy X-ray absorptiometry. The following categorical variables were included in our analysis as covariates: race and moderate activities. The continuous covariates in our study were as follows: age, body mass index, the family income-to-poverty ratio, and blood urea nitrogen, serum uric acid, total protein, total cholesterol, serum phosphorus, and serum calcium levels. The family income-to-poverty ratio was calculated by dividing the annual family income by the poverty guideline. More information on testosterone, estradiol, SHBG, total BMD and all covariates can be found at https://www.cdc.gov/nchs/nhanes/.

### Statistical Analyses

All analyses were calculated based on the weights of the NHANES samples. The participants in the study were divided into quartiles based on testosterone, estradiol or SHBG levels. In accordance with the Strengthening the Reporting of Observational Studies in Epidemiology (STROBE) statement (10), we developed three models: Model 1: no covariates were adjusted; Model 2: age and race were adjusted; and Model 3: all covariates were adjusted. We assessed the independent association of testosterone, estradiol, and SHBG with total BMD separately using weighted multiple linear regression models. We revealed underlying nonlinear associations by using weighted generalized additive models and smooth curve fitting. In addition, we used a two-piecewise linear regression model to calculate the threshold effects when nonlinear associations existed.

The continuous and categorical variables are presented as the mean ± standard deviation and percentage, respectively. The statistical significance was set at P<0.05 for this study. All statistical analyses were carried out by EmpowerStats software (http://www.empowerstats.com) and R software (version 3.4.3).

## Results

**Tables 1**, **2** show the descriptions of the sociodemographic and medical characteristics of boys and girls, respectively. In **Table 1**, we divided the testosterone, estradiol and SHBG levels of 853 boys into quartiles (Q1-Q4; from Q1 to Q4, the levels gradually increase). For testosterone levels, boys with higher testosterone levels had higher levels of total BMD than boys in the Q1 group. For estradiol levels, from Q1 to Q4, estradiol levels increased, with a corresponding increase in total BMD levels. However, this trend was reversed for SHBG levels, and boys with higher SHBG levels had lower total BMD levels. In **Table 2**, the testosterone, estradiol and SHBG levels of 795 girls were divided into quartiles. For testosterone levels, girls with higher testosterone levels had higher total BMD levels. For estradiol levels, girls in the Q2 group had the lowest total BMD. Among the SHBG level groups, the total BMD level was lowest in the Q3 group.

**Table 1:** Descriptions of 853 boys included in the present study.

	Testosterone					Estradiol					Sex hormone-binding globulin					
	Q1	Q2	Q3	Q4	P value	Q1	Q2	Q3	Q4	P value		Q1	Q2	Q3	Q4	P value
Age (years)	13.42 ± 1.58	15.52 ± 2.09	16.27 ± 2.01	16.12 ± 1.92	<0.0001	13.19 ± 1.38	15.11 ± 1.87	16.27 ± 2.00	16.82 ± 1.66	<0.0001	Age (years)	16.36 ± 2.11	15.95 ± 1.99	15.15 ± 1.90	13.96 ± 2.07	<0.0001
Race/Ethnicity (%)					0.7371					0.1357	Race/Ethnicity (%)					<0.0001
Mexican American	12.19	18.13	18.62	15.73		12.21	15.35	19.64	17.78		Mexican American	25.03	18.62	12.42	9.82	
Other Hispanic	8.72	6.02	6.00	9.14		7.72	6.17	8.34	7.78		Other Hispanic	9.41	9.26	5.63	5.87	
Non-Hispanic White	57.46	57.86	55.57	52.49		61.48	61.43	49.80	49.77		Non-Hispanic White	44.42	52.78	56.75	67.35	
Non-Hispanic Black	13.14	9.91	10.92	13.90		12.97	10.24	11.22	13.45		Non-Hispanic Black	9.77	10.09	15.50	12.34	
Other Race/Ethnicity	8.50	8.07	8.88	8.74		5.61	6.81	10.99	11.23		Other Race/Ethnicity	11.37	9.25	9.69	4.62	
Body mass index (kg/m²)	23.86 ± 7.16	25.59 ± 7.70	24.15 ± 4.94	22.14 ± 3.60	<0.0001	21.23 ± 4.70	23.16 ± 5.61	25.63 ± 7.03	26.27 ± 6.37	<0.0001	Body mass index (kg/m²)	30.36 ± 6.86	24.47 ± 5.36	22.09 ± 3.82	19.95 ± 3.37	<0.0001
Income to poverty ratio	4.75 ± 1.79	4.93 ± 3.68	4.91 ± 2.62	5.15 ± 5.43	0.7277	4.81 ± 1.81	4.84 ± 3.50	5.10 ± 4.79	5.00 ± 3.88	0.8183	Income to poverty ratio	4.94 ± 2.59	4.73 ± 2.93	5.28 ± 5.41	4.81 ± 2.91	0.4334
Moderate activities (%)					0.1443					0.0014	Moderate activities (%)					0.6156
Yes	59.08	58.54	56.35	55.27		56.99	63.67	49.74	58.31		Yes	55.43	55.83	61.46	56.90	
No	38.31	39.85	43.32	44.73		39.61	35.77	49.61	41.69		No	43.14	43.93	36.94	41.62	
Not recorded	2.61	1.61	0.33			3.39	0.56	0.65			Not recorded	1.43	0.24	1.60	1.48	
Blood urea nitrogen (mg/dL)	11.86 ± 3.63	12.32 ± 3.48	11.92 ± 3.08	11.77 ± 3.74	0.3781	12.08 ± 3.48	12.20 ± 3.54	11.62 ± 3.55	11.96 ± 3.41	0.3641	Blood urea nitrogen (mg/dL)	11.64 ± 3.23	12.20 ± 3.43	12.46 ± 3.83	11.63 ± 3.43	0.0310
Serum uric acid (mg/dL)	5.18 ± 1.30	5.83 ± 1.33	5.83 ± 1.03	5.60 ± 0.97	<0.0001	4.77 ± 1.05	5.70 ± 1.12	5.96 ± 1.04	6.05 ± 1.16	<0.0001	Serum uric acid (mg/dL)	6.47 ± 1.16	5.78 ± 0.97	5.51 ± 1.07	4.83 ± 1.01	<0.0001
Total protein (g/L)	71.40 ± 4.16	72.16 ± 4.29	72.99 ± 4.37	73.29 ± 4.35	<0.0001	70.78 ± 4.27	72.45 ± 4.32	72.81 ± 4.06	73.82 ± 4.16	<0.0001	Total protein (g/L)	73.36 ± 4.30	72.95 ± 4.38	72.53 ± 4.33	71.12 ± 4.07	<0.0001
Total cholesterol (mg/dL)	155.75 ± 31.15	155.53 ± 29.27	153.24 ± 28.10	154.98 ± 28.85	0.8190	154.01 ± 30.00	150.38 ± 26.92	154.50 ± 28.79	161.42 ± 30.94	0.0013	Total cholesterol (mg/dL)	162.22 ± 33.74	153.26 ± 28.94	150.59 ± 24.46	154.14 ± 28.89	0.0006
Serum phosphorus (mg/dL)	4.94 ± 0.65	4.45 ± 0.67	4.25 ± 0.75	4.28 ± 0.61	<0.0001	5.03 ± 0.62	4.54 ± 0.67	4.28 ± 0.64	4.07 ± 0.57	<0.0001	Serum phosphorus (mg/dL)	4.19 ± 0.58	4.36 ± 0.68	4.55 ± 0.75	4.81 ± 0.71	<0.0001
Serum calcium (mg/dL)	9.61 ± 0.31	9.66 ± 0.29	9.68 ± 0.29	9.69 ± 0.29	0.0115	9.63 ± 0.28	9.67 ± 0.30	9.62 ± 0.32	9.72 ± 0.28	0.0047	Serum calcium (mg/dL)	9.67 ± 0.32	9.66 ± 0.29	9.65 ± 0.31	9.66 ± 0.27	0.9330
Total BMD (g/cm²)	0.94 ± 0.12	1.04 ± 0.13	1.07 ± 0.12	1.09 ± 0.12	<0.0001	0.91 ± 0.10	1.03 ± 0.12	1.09 ± 0.11	1.11 ± 0.11	<0.0001	Total BMD (g/cm²)	1.09 ± 0.11	1.08 ± 0.12	1.02 ± 0.12	0.94 ± 0.13	<0.0001

Mean ± SD for continuous variables: P-value was calculated by weighted linear regression model. % for categorical variables: P-value was calculated by weighted chi-square test.

**Table 2:** Descriptions of 795 girls included in the present study.

	Testosterone					Estradiol					Sex hormone-binding globulin					
	Q1	Q2	Q3	Q4	P value	Q1	Q2	Q3	Q4	P value		Q1	Q2	Q3	Q4	P value
Age (years)	14.65 ± 2.19	15.28 ± 2.07	15.44 ± 2.10	15.86 ± 1.98	<0.0001	15.23 ± 2.31	14.95 ± 2.13	15.30 ± 2.04	15.85 ± 1.87	0.0004	Age (years)	15.35 ± 2.23	15.35 ± 2.01	15.20 ± 2.26	15.41 ± 2.02	0.7709
Race/Ethnicity (%)					0.8543					0.0075	Race/Ethnicity (%)					<0.0001
Mexican American	15.18	17.67	14.99	12.20		11.42	17.25	12.57	19.24		Mexican American	25.57	13.81	17.74	5.59	
Other Hispanic	10.59	9.36	6.46	7.94		4.46	10.29	13.20	6.83		Other Hispanic	9.94	11.54	5.84	7.19	
Non-Hispanic White	53.97	54.99	56.55	56.00		66.19	52.36	51.65	49.47		Non-Hispanic White	43.75	49.07	53.31	71.09	
Non-Hispanic Black	10.79	10.53	10.52	12.86		8.09	11.94	11.58	13.77		Non-Hispanic Black	9.88	11.69	13.59	9.79	
Other Race/Ethnicity	9.47	7.46	11.47	11.00		9.85	8.17	11.00	10.68		Other Race/Ethnicity	10.86	13.90	9.52	6.35	
Body mass index (kg/m²)	23.75 ± 5.65	23.81 ± 5.57	25.50 ± 7.15	25.14 ± 5.71	0.0050	24.46 ± 5.61	24.87 ± 6.69	24.75 ± 6.15	24.31 ± 6.12	0.7984	Body mass index (kg/m²)	29.98 ± 6.94	25.29 ± 4.94	22.23 ± 4.19	21.98 ± 4.85	<0.0001
Income to poverty ratio	5.42 ± 6.79	5.63 ± 6.32	4.65 ± 1.82	4.69 ± 3.56	0.1077	4.75 ± 3.96	5.45 ± 7.33	5.31 ± 4.96	4.85 ± 2.40	0.4134	Income to poverty ratio	6.25 ± 7.93	4.80 ± 3.79	5.03 ± 4.16	4.45 ± 2.98	0.0025
Moderate activities (%)					0.0164					<0.0001	Moderate activities (%)					0.0267
Yes	56.69	54.11	56.38	52.33		65.11	56.86	45.96	49.41		Yes	52.62	59.69	53.67	53.58	
No	42.74	45.22	39.94	47.67		34.89	42.12	49.91	50.37		No	46.77	40.31	42.68	45.60	
Not recorded	0.58	0.66	3.68				1.02	4.13	0.22		Not recorded	0.61		3.65	0.82	
Blood urea nitrogen (mg/dL)	162.04 ± 26.82	10.75 ± 3.40	10.65 ± 4.94	10.29 ± 2.72	0.2949	10.74 ± 3.00	10.85 ± 5.11	10.04 ± 2.80	10.18 ± 3.18	0.0649	Blood urea nitrogen (mg/dL)	9.88 ± 2.55	10.66 ± 2.67	10.58 ± 3.38	10.65 ± 4.92	0.1106
Serum uric acid (mg/dL)	4.49 ± 1.02	4.33 ± 1.07	4.63 ± 1.07	4.41 ± 1.04	0.0249	4.40 ± 0.97	4.68 ± 1.24	4.43 ± 0.94	4.37 ± 1.04	0.0157	Serum uric acid (mg/dL)	5.20 ± 1.18	4.41 ± 0.97	4.27 ± 0.86	4.14 ± 0.91	<0.0001
Total protein (g/L)	162.04 ± 26.82	158.57 ± 29.32	158.60 ± 29.11	164.69 ± 30.73	0.0970	163.93 ± 28.82	161.98 ± 29.23	161.37 ± 29.80	156.04 ± 28.43	0.0463	Total protein (g/L)	165.54 ± 32.69	155.81 ± 26.67	155.91 ± 25.26	165.94 ± 30.04	<0.0001
Total cholesterol (mg/dL)	71.45 ± 3.76	71.98 ± 3.97	72.37 ± 4.33	71.76 ± 4.01	0.1392	71.80 ± 3.75	72.27 ± 4.06	72.02 ± 4.56	71.56 ± 3.79	0.3584	Total cholesterol (mg/dL)	72.30 ± 4.08	72.61 ± 4.03	72.15 ± 3.76	70.85 ± 4.06	<0.0001
Serum phosphorus (mg/dL)	4.39 ± 0.62	4.26 ± 0.56	4.18 ± 0.55	4.16 ± 0.52	0.0003	4.28 ± 0.61	4.35 ± 0.59	4.19 ± 0.53	4.12 ± 0.50	0.0004	Serum phosphorus (mg/dL)	4.20 ± 0.63	4.19 ± 0.51	4.32 ± 0.54	4.25 ± 0.59	0.1179
Serum calcium (mg/dL)	9.56 ± 0.32	9.53 ± 0.27	9.54 ± 0.32	9.52 ± 0.29	0.6382	9.55 ± 0.34	9.56 ± 0.27	9.55 ± 0.28	9.50 ± 0.31	0.2507	Serum calcium (mg/dL)	9.56 ± 0.30	9.50 ± 0.27	9.60 ± 0.27	9.50 ± 0.34	0.0009
Total BMD (g/cm²)	0.98 ± 0.11	1.02 ± 0.10	1.03 ± 0.10	1.04 ± 0.10	<0.0001	1.01 ± 0.12	1.00 ± 0.10	1.02 ± 0.10	1.04 ± 0.10	0.0023	Total BMD (g/cm²)	1.04 ± 0.11	1.04 ± 0.10	1.00 ± 0.10	1.01 ± 0.10	<0.0001

Mean ± SD for continuous variables: P-value was calculated by weighted linear regression model. % for categorical variables: P-value was.

### Association Between Testosterone and Total BMD

**Figure 2:**
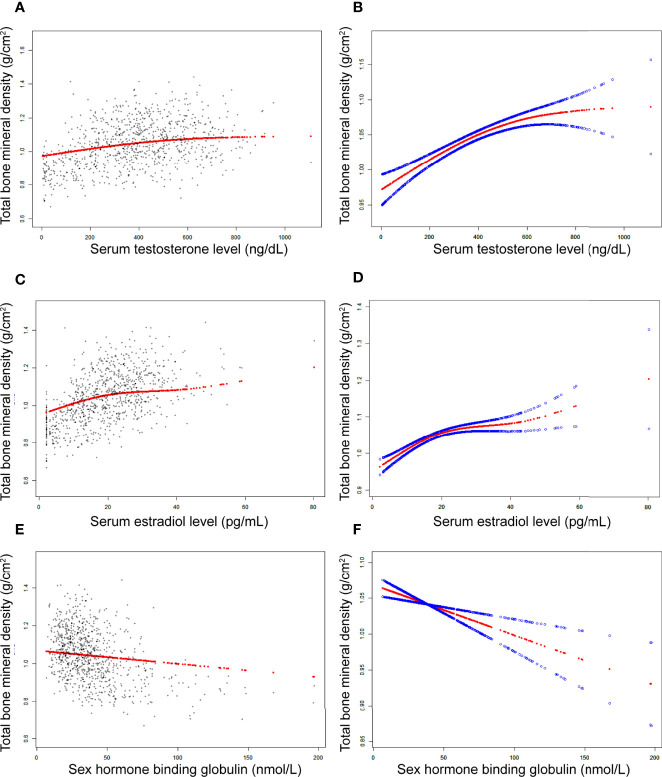
The association between testosterone **(A, B)**, estradiol **(C, D)**, and SHBG **(E, F)** and total bone mineral density in boys, respectively. **(A, C, E)** Each black point represents a sample. **(B, D, F)** Solid red line represents the smooth curve fit between variables. Blue bands represent the 95% of confidence interval from the fit. Adjusted for age, race, body mass index, ratio of family income to poverty, moderate activities, blood urea nitrogen, serum uric acid, total protein, total cholesterol, serum phosphorus, and serum calcium.

In boys (**Table 3**; **Figure 2**), the association between testosterone and total BMD was positive in all three regression models (**Table 3**): Model 1 [0.0003 (0.0002, 0.0003)]; Model 2 [0.0001 (0.0000, 0.0001)]; and Model 3 [0.0001 (0.0001, 0.0002)]. The P values were all statistically significant (P<0.001).

**Table 3:** Association of serum testosterone, estradiol, and sex hormone-binding globulin levels with total bone mineral density in boys.

	Model 1 β (95% CI)	Model 2 β (95% CI)	Model 3 β (95% CI)
Testosterone	0.0003 (0.0002, 0.0003) <0.000001	0.0001 (0.0000, 0.0001) 0.000035	0.0001 (0.0001, 0.0002) <0.000001
Testosterone categories			
Q1	0	0	0
Q2	0.0980 (0.0751, 0.1209) <0.000001	0.0277 (0.0071, 0.0483) 0.008421	0.0273 (0.0078, 0.0468) 0.006311
Q3	0.1322 (0.1086, 0.1558) <0.000001	0.0350 (0.0128, 0.0573) 0.002100	0.0494 (0.0279, 0.0709) 0.000008
Q4	0.1454 (0.1218, 0.1689) <0.000001	0.0513 (0.0294, 0.0732) 0.000005	0.0788 (0.0571, 0.1006) <0.000001
P for trend	<0.001	<0.001	<0.001
Estradiol	0.0070 (0.0063, 0.0078) <0.000001	0.0032 (0.0024, 0.0040) <0.000001	0.0029 (0.0021, 0.0037) <0.000001
Estradiol categories			
Q1	0	0	0
Q2	0.1125 (0.0917, 0.1333) <0.000001	0.0603 (0.0403, 0.0802) <0.000001	0.0563 (0.0361, 0.0764) <0.000001
Q3	0.1784 (0.1569, 0.1999) <0.000001	0.0922 (0.0696, 0.1147) <0.000001	0.0852 (0.0622, 0.1082) <0.000001
Q4	0.1959 (0.1745, 0.2173) <0.000001	0.0929 (0.0692, 0.1166) <0.000001	0.0833 (0.0588, 0.1077) <0.000001
P for trend	<0.001	<0.001	<0.001
Sex hormone-binding globulin	-0.0024 (-0.0027, -0.0021) <0.000001	-0.0011 (-0.0015, -0.0008) <0.000001	-0.0007 (-0.0011, -0.0004) 0.000072
Sex hormone-binding globulin categories			
Q1	0	0	0
Q2	-0.0056 (-0.0294, 0.0181) 0.642019	0.0080 (-0.0117, 0.0277) 0.425053	0.0312 (0.0104, 0.0520) 0.003420
Q3	-0.0648 (-0.0889, -0.0408) <0.000001	-0.0278 (-0.0482, -0.0074) 0.007671	0.0063 (-0.0166, 0.0293) 0.587642
Q4	-0.1451 (-0.1682, -0.1220) <0.000001	-0.0662 (-0.0872, -0.0452) <0.000001	-0.0210 (-0.0463, 0.0042) 0.102539
P for trend	<0.001	<0.001	0.008

Model 1, no covariates were adjusted. Model 2, age, race were adjusted. Model 3, age, race, body mass index, ratio of family income to poverty, moderate activities, blood urea nitrogen, serum uric acid, total protein, total cholesterol, serum phosphorus, and serum calcium were adjusted.

**Figure 3:**
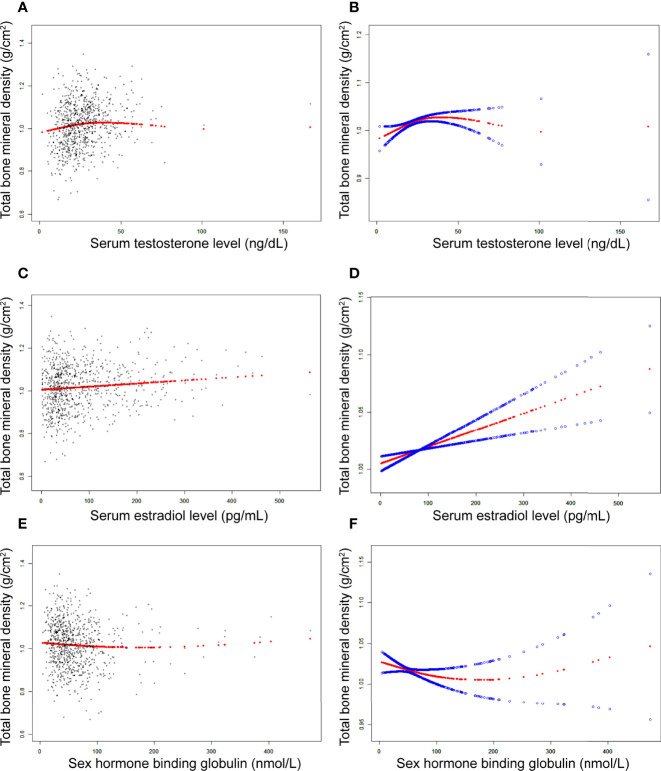
The association between testosterone **(A, B)**, estradiol **(C, D)**, and SHBG **(E, F)** and total bone mineral density in girls, respectively. **(A, C, E)** Each black point represents a sample. **(B, D, F)**, Solid red line represents the smooth curve fit between variables. Blue bands represent the 95% of confidence interval from the fit. Adjusted for age, race, body mass index, ratio of family income to poverty, moderate activities, blood urea nitrogen, serum uric acid, total protein, total cholesterol, serum phosphorus, and serum calcium.

In girls (**Table 4**; **Figure 3**), the association of testosterone with total BMD was positive in Model 1 [0.0014 (0.0008, 0.0019)] and Model 2 [0.0006 (0.0000, 0.0011)], whereas in Model 3, the association was not significant [0.0004 (-0.0001, 0.0008)] (**Table 4**). The P values for all three models were statistically significant (P < 0.05). The nonlinear association between testosterone and total BMD is presented in **Figure 3B**. The inflection point of the inverted U-shaped curve between testosterone and total BMD was calculated to be a testosterone level of 25.4 ng/dL by using a two-piecewise linear regression model (**Table 5**).

**Table 4:** Association of serum testosterone, estradiol, and sex hormone-binding globulin levels with total bone mineral density in girls.

	Model 1 β (95% CI)	Model 2 β (95% CI)	Model 3 β (95% CI)
Testosterone	0.0014 (0.0008, 0.0019) 0.000001	0.0006 (0.0000, 0.0011) 0.032395	0.0004 (-0.0001, 0.0008) 0.129161
Testosterone categories			
Q1	0	0	0
Q2	0.0317 (0.0108, 0.0526) 0.003052	0.0183 (-0.0003, 0.0370) 0.054683	0.0153 (-0.0020, 0.0326) 0.084278
Q3	0.0495 (0.0291, 0.0699) 0.000002	0.0320 (0.0137, 0.0504) 0.000655	0.0203 (0.0031, 0.0375) 0.020724
Q4	0.0546 (0.0341, 0.0751) <0.000001	0.0268 (0.0082, 0.0455) 0.004953	0.0210 (0.0038, 0.0383) 0.017147
P for trend	<0.001	0.002	0.017
Estradiol	0.0002 (0.0001, 0.0003) 0.000058	0.0001 (0.0000, 0.0002) 0.004228	0.0001 (0.0001, 0.0002) 0.000257
Estradiol categories			
Q1	0	0	0
Q2	-0.0066 (-0.0267, 0.0135) 0.521140	-0.0001 (-0.0180, 0.0178) 0.990773	0.0017 (-0.0149, 0.0182) 0.845114
Q3	0.0116 (-0.0085, 0.0317) 0.258851	0.0105 (-0.0074, 0.0285) 0.250676	0.0112 (-0.0055, 0.0280) 0.189114
Q4	0.0312 (0.0111, 0.0513) 0.002435	0.0167 (-0.0013, 0.0347) 0.069677	0.0189 (0.0022, 0.0357) 0.027236
P for trend	<0.001	0.041	0.017
Sex hormone-binding globulin	-0.0001 (-0.0002, 0.0000) 0.133284	-0.0002 (-0.0003, -0.0001) 0.000132	-0.0001 (-0.0002, 0.0001) 0.242211
Sex hormone-binding globulin categories			
Q1	0	0	0
Q2	0.0054 (-0.0159, 0.0267) 0.620522	0.0026 (-0.0161, 0.0213) 0.784763	0.0105 (-0.0085, 0.0295) 0.277503
Q3	-0.0340 (-0.0551, -0.0129) 0.001645	-0.0354 (-0.0539, -0.0169) 0.000191	-0.0085 (-0.0285, 0.0115) 0.405766
Q4	-0.0314 (-0.0517, -0.0112) 0.002442	-0.0384 (-0.0566, -0.0202) 0.000038	-0.0075 (-0.0281, 0.0131) 0.476320
P for trend	<0.001	<0.001	0.160

Model 1, no covariates were adjusted. Model 2, age, race were adjusted. Model 3, age, race, body mass index, ratio of family income to poverty, moderate activities, blood urea nitrogen, serum uric acid, total protein, total cholesterol, serum phosphorus, and serum calcium were adjusted.

**Table 5:** Threshold effect analysis of serum testosterone level on total bone mineral density using two-piecewise linear regression model.

Total bone mineral density	Adjusted β (95% CI), P value
Serum testosterone level	
Fitting by standard linear model	0.0004 (-0.0001, 0.0008) 0.129161
Fitting by two-piecewise linear model	
Inflection point	25.4 (ng/dL)
Serum testosterone level < 25.4 (ng/dL)	0.0021 (0.0009, 0.0034) 0.0008
Serum testosterone level > 25.4 (ng/dL)	-0.0003 (-0.0010, 0.0003) 0.3095
Log likelihood ratio	0.002

Age, race, body mass index, ratio of family income to poverty, moderate activities, blood urea nitrogen, serum uric acid, total protein, total cholesterol, serum phosphorus, and serum calcium were adjusted.

### Association Between Estradiol and Total BMD

In boys (**Table 3**; **Figure 2**), estradiol was positively associated with total BMD in all three models (**Table 3**): Model 1 [0.0070 (0.0063, 0.0078)]; Model 2 [0.0032 (0.0024, 0.0040)]; and Model 3 [0.0029 (0.0021, 0.0037)]. The P values were all statistically significant (P<0.001). There was a specific point between estradiol and total BMD (24.3 pg/mL of estradiol), after which the positive correlation between estradiol and total BMD was relatively insignificant (**Figure 2D**).

In girls (**Table 4**; **Figure 3**), the association between estradiol and total BMD was positive in all three models (**Table 4**): Model 1 [0.0002 (0.0001, 0.0003)]; Model 2 [0.0001 (0.0000, 0.0002)]; and Model 3 [0.0001 (0.0001, 0.0002)] (P for trend < 0.05 for each).

### Association Between SHBG and Total BMD

In boys (**Table 3**; **Figure 2**), SHBG was negatively associated with total BMD in all three regression models (**Table 3**): Model 1 [-0.0024 (-0.0027, -0.0021)]; Model 2 [-0.0011 (-0.0015, -0.0008)]; and Model 3 [-0.0007 (-0.0011, -0.0004)] (P for trend < 0.01 for each).

In girls (**Table 4**; **Figure 3**), we did not find a significant correlation between SHBG and total BMD (**Table 4**): Model 1 [-0.0001 (-0.0002, 0.0000)]; Model 3 [-0.0001 (-0.0002, 0.0001)]. The P value for Model 3 was not significant (P>0.05).

## Discussion

The population of adolescent boys and girls selected for our study was nationally representative. The association of sex hormones with total BMD differed in boys and girls. In boys, testosterone and estradiol levels were positively associated with total BMD, whereas SHBG levels were negatively associated with total BMD. In girls, estradiol was positively associated with total BMD, whereas testosterone and SHBG levels were not significantly associated with total BMD. Notably, we found that in boys, there was a point between estradiol and total BMD (24.3 pg/mL of estradiol), after which the positive correlation between estradiol and total BMD was relatively insignificant. In girls, there was an inverted U-shaped association between total BMD and testosterone with the inflection point at 25.4 ng/dL of testosterone.

A cross-sectional study of 1070 Korean men reported that both total testosterone and free testosterone were positively correlated with BMD, and genetic effects played an important part in the association of testosterone with BMD (11). A cross-sectional study of adults aged 40-60 years reported that testosterone levels were negatively associated with total BMD (12). Furthermore, total testosterone levels were not significantly associated with total BMD in adolescent girls. However, we found that too much testosterone can not only cause virilization and infertility but also reduce BMD and affect bone health in women. However, we need more prospective intervention studies to support our findings.

A growing number of studies have reported that decreased estradiol levels are associated with decreased BMD. A study of postmenopausal women with and without osteoporosis found that serum estradiol levels were markedly lower in those with osteoporosis than in those without osteoporosis, suggesting that there was a positive association between estradiol and BMD (13). A previous study of male college athletes found that free and total estradiol levels were important positive determinants of BMD (14). Moreover, a previous genome-wide study confirmed the role of estradiol on BMD and bone health in men and women (15). In our study, we found that the relationship between estradiol and total BMD in boys was not always significantly positive.

A cross-sectional study of US adults reported that the value of SHBG for predicting bone loss in adults may improve (7). A previous cross-sectional study of 142 Moroccan men with no prior diagnosis of osteoporosis reported that BMD at the total hip was negatively correlated with SHBG (16). A study of Chinese men over 45 years old reported a negative correlation between serum SHBG levels and BMD (17). Additionally, a study of premenopausal women reported a negative relationship between SHBG levels and bone mass (18). The evidence mentioned above suggests that higher SHBG levels may play a significant role in the development of osteoporosis. In our study, this relationship was not significant in girls.

Our data were obtained from the NHANES, and NHANES data were acquired according to standard protocols, which ensures that our findings are consistent and accurate. However, we should clearly recognize the limitations of our study. First, the NHANES is a large cross-sectional survey and therefore cannot determine causal relationships between exposure factors and outcome variables, and more cohort studies are needed to confirm our conclusions. Second, the samples in the NHANES were only assessed once, and some data were missing, which may lead to potential bias. Therefore, it is recommended that further studies are required to perform multiple tests. Third, although we used nationally representative population data, the study was limited to adolescent boys and girls aged 12–19 years. Consequently, the study’s conclusions may not be applicable to children and other populations.

## Conclusion

We found differences in the association of sex hormones with total BMD in boys and girls. An inverted U-shaped association between testosterone levels and total BMD in girls with the inflection point at 25.4 ng/dL of testosterone suggests that an appropriate increase in serum testosterone levels may be beneficial for skeletal development, whereas a high testosterone level may be detrimental to BMD. Furthermore, keeping estradiol levels below a certain level in boys (24.3 pg/mL) may be considered.

## Data Availability Statement

The original contributions presented in the study are included in the article/**Supplementary Material**. Further inquiries can be directed to the corresponding author.

## Author Contributions

KX and MZ conceptualized and designed the study. KX collected data, performed data analysis, and participated in the writing the manuscript. KX and YF revised the manuscript. KX and MZ reviewed the manuscript. KX, YF and BC produced figures and tables. All authors approved the final manuscript and agreed to be responsible for all aspects of the study.

## Conflict of Interest

The authors declare that the research was conducted in the absence of any commercial or financial relationships that could be construed as a potential conflict of interest.

## Publisher’s Note

All claims expressed in this article are solely those of the authors and do not necessarily represent those of their affiliated organizations, or those of the publisher, the editors and the reviewers. Any product that may be evaluated in this article, or claim that may be made by its manufacturer, is not guaranteed or endorsed by the publisher.
